# The Keystone Veterinary Medical Association

**Published:** 1890-06

**Authors:** W. H. Ridge

**Affiliations:** Secretary


### SOCIETY PROCEEDINGS.

*The Keystone Veterinary Medical Association.*—This Association held its monthly meeting at the College of Physicians, Philadelphia, May 3d, 1890. Dr. Huidekoper presided. The following members were present: Drs. Hoskins, Huidekoper, Weber, Williams, Webster, Harger, Ridge, Lusson, Magill, Werntz, Mattson. Dr. Schreiber was present as a guest.

The minutes of the April meeting were read and approved. Dr. Miller and Dr. Hartman, essayists for the evening, were absent. The Secretary read a communication from Dr. Miller, stating that he was unexpectedly called from the city. Dr. Huidekoper read a paper on fever, which he defined as "a general condition of the animal body in which there is an elevation of the animal body temperature, which may be only a degree or two or may be ten degrees Farenheit. The elevation of the body temperature, which represents tissue change or combustion, is accompanied by an acceleration of the heart's action, a quickening of the respiration and an aberration of the functional activity of the various organs of the body. These organs may be stimulated to the performance of excessive work, or they may be incapacitated from carrying out their allotted tasks, or in the course of a fever the two conditions may both exist, the one succeeding the other. To fever as a disease, is usually added chills as an essential symptom. * * *

"Essential fevers are subdivided into *ephemeral fevers*, which last but a short time and terminate by critical phenomena; *intermittent fevers*, in which there are alternations of exacerbations of the febrile symptoms and remissions, in which the body returns to its normal condition or sometimes to a depressed condition, in which the functions of life are but badly performed; and, *continued fevers*, which include the contagious diseases, as glanders, influenza, etc., the septic diseases, as pyæmia, septicæmia, etc., and the *eruplive fevers*, as variola, etc. * * *

"There are times when it is difficult to distinguish between the existence of fever as a disease and a temporary feverish condition which is the result of excessive work. Like the condition of congestion of the lungs, which is normal up to a certain degree, as in the lungs of a race horse after a severe race, and morbid when it produces more than temporary phenomena, or when it causes distinct lesions, fever, or it is better termed a feverish condition, may follow any work or other employment of energy in which excessive tissue change has taken place, but if the consequences are ephemeral and no recognizable lesion is apparent, it is not considered morbid. * * * Fever, with its symptoms of increased temperature, acceleration of the pulse, acceleration of the respiration, dry skin, diminished secretion, etc., must be considered as a symptom of organic disturbance. This organic disturbance may be the result of *local inflammation* or other irritant acting through the nerves on nerve centres; *alteration of the blood*, in which a poison is carried to the nerve centres, or *direct irritants* to the nerve centres themselves, as in cases of heat stroke, injuries to the brain, etc."

The paper included the symptoms of fever as it occurs in the horse, and outlined the principles of treatment for the various forms of the disease.

Dr. Williams reported a case in a three year old which always had been a spare eater. It had a tucked-up look and was hide-bound. It had had five short attacks of colic within one month. The last attack had the following symptoms: Sweating, pawing, looking around at side; did not struggle much; had regurgitation of gas; nausea was very marked. Dr Williams punctured the cæcum and got only a small quantity of gas. Autopsy: Intestines normal, except stomach, which was greatly distended; on opening it found a large quantity of coffee-colored fluid; at pyloric end found a large ulcer, which was so œdematous that it obstructed the entrance to duodenum. In the cardiac end the mucous membrane was thick and had several ruptures. He thought this was due to the great distention of the organ. There were a large number of bots in duodenum.

Dr. Huidekoper thought ulcerative gastritis rare in the horse.

Dr. Hoskins reported some cases which at first he thought were influenza. Temperature 103° F.; pulse 45; respiration normal; off front leg and near hind leg swollen. After a laxative, in ten days he found an ulcer on front leg; in five days more found ulcers in nostril; this extended to three other cases; one he had at his own stable. Diagnosis, glanders; had them destroyed. Had his stall thoroughly disinfected, then put his own driving horse in same stall; in about a week it became sluggish. Temperature never lower than 101 2-5° to 103° F. There soon appeared a circumscribed swelling on the side, which seemed to fluctuate; on opening found a sticky serum; ulcer refuses to heal. Gave aloes and calomel.

The horse became lame. It has had bog spavins, but one has entirely disappeared since its sickness; there is no discharge from the nose. Dr. Hoskins asked if it was proper to drive this animal on the streets. Dr. Huidekoper stated that as there are no laws prohibiting the use of a glandered animal in Pennsylvania he thought that the bringing of glandered animals into this State should be encouraged, so as to awaken our law-makers out of their lethargic state and force them to pass laws. Dr. Williams reported a case where a man is driving a glandered horse every day in this city, and knows of at least one animal that has been infected from it.

Dr. Hoskins reported a case of a cribber which fractured the pre-maxillary bone anterior to tusks; on trying to replace it he found that he could not get it back to its proper place. Every day he pressed on incisors, when, little by little, they regained their proper position, the upper lip being their only bandage. Dr. Huidekoper reported a five year old Clydesdale, not eating, emaciated, as it was changing its teeth. He thought the emaciation was due to this cause, but on watching it found that it would hold its head high in the air and suck wind in a position that the animal rarely assumes to crib. Dr. Werntz reported a case in which the horse had not eaten for five days; pulse, respiration and temperature normal. Mucous membrane rosy. He could not find anything on which to base a diagnosis. Thinking of a similar case, he examined the mouth, when he found a corncob wedged in between the two rows of molars; on removal the horse began to eat. Dr. Huidekoper reported a case of anorexia due to a nail between the teeth.

Dr. Williams reported one in which a split molar was the cause.

Dr. Weber gave the legislative committee a report to be read at the next meeting.

W. H. Ridge, *Secretary.*

